# Modelling the household-level impact of a maternal respiratory syncytial virus (RSV) vaccine in a high-income setting

**DOI:** 10.1186/s12916-020-01783-8

**Published:** 2020-11-12

**Authors:** Patricia T. Campbell, Nicholas Geard, Alexandra B. Hogan

**Affiliations:** 1 Epidemiology, University of Melbourne, at the Peter Doherty Institute for Infection and Immunity, Melbourne, Victoria Australia; 2 School of Population and Global Health, The University of Melbourne, Melbourne, Australia; 3 School of Computing and Information Systems, Melbourne School of Engineering, The University of Melbourne, Melbourne, Australia; 4 MRC Centre for Global Infectious Disease Analysis, Department of Infectious Disease Epidemiology, School of Public Health, Faculty of Medicine, Imperial College London, London, UK

**Keywords:** Respiratory syncytial virus, Maternal vaccine, Individual-based model, Mathematical modelling, Transmission

## Abstract

**Background:**

Respiratory syncytial virus (RSV) infects almost all children by the age of 2 years, with the risk of hospitalisation highest in the first 6 months of life. Development and licensure of a vaccine to prevent severe RSV illness in infants is a public health priority. A recent phase 3 clinical trial estimated the efficacy of maternal vaccination at 39% over the first 90 days of life. Households play a key role in RSV transmission; however, few estimates of population-level RSV vaccine impact account for household structure.

**Methods:**

We simulated RSV transmission within a stochastic, individual-based model framework, using an existing demographic model, structured by age and household and parameterised with Australian data, as an exemplar of a high-income country. We modelled vaccination by immunising pregnant women and explicitly linked the immune status of each mother-infant pair. We quantified the impact on children for a range of vaccine properties and uptake levels.

**Results:**

We found that a maternal immunisation strategy would have the most substantial impact in infants younger than 3 months, reducing RSV infection incidence in this age group by 16.6% at 70% vaccination coverage. In children aged 3–6 months, RSV infection was reduced by 5.3%. Over the first 6 months of life, the incidence rate for infants born to unvaccinated mothers was 1.26 times that of infants born to vaccinated mothers. The impact in older age groups was more modest, with evidence of infections being delayed to the second year of life.

**Conclusions:**

Our findings show that while individual benefit from maternal RSV vaccination could be substantial, population-level reductions may be more modest. Vaccination impact was sensitive to the extent that vaccination prevented infection, highlighting the need for more vaccine trial data.

## Background

Respiratory syncytial virus (RSV) causes respiratory illness in young children and presents a substantial global public health burden, with almost all children being infected before the age of 2 years. In 2015, the global incidence of RSV in children younger than 5 years was estimated as 33.1 million, with 3.2 million hospitalisations in that age group [1].

There is no approved vaccine for RSV, but the World Health Organization (WHO) has identified the development of a vaccine for RSV as a key priority [2, 3]. Clinical trials are progressing, with at least 19 vaccine candidates in phase 1–3 trials, and additional products in preclinical development [4]. The key target groups for RSV immunisation are pregnant women, infants, young children, and the elderly, although vaccination of pregnant women (maternal immunisation) in the third trimester of pregnancy is currently the most imminent strategy and, operationally, could be aligned with existing prenatal health system contacts [4, 5]. Maternal immunisation aims to elicit high levels of protective RSV-specific antibody in pregnant women, conferring immunity via transplacental transfer of antibodies to the unborn infant, and fostering protection from RSV disease in the first few months of life, when the risk of hospitalisation from severe RSV disease is highest [6–8]. Topline results for a large-scale multi-country phase 3 clinical trial for the maternal vaccine candidate ResVax (Novavax) were recently announced. While the primary efficacy endpoint of efficacy against medically significant RSV lower respiratory tract infection (LRTI) through 90 days was not met (39.4%, 95% CI 5.3–61.2%), efficacy was demonstrated in preventing RSV LRTI hospitalisation through 90 days (44.4%, 95% CI 19.6–61.5%) and all-cause respiratory illness-related hospitalisation through 180 days (25.3%, 95% CI 5.3–41.0%) [9, 10].

Early trial results suggest RSV vaccines will be relatively short-lasting and unlikely to produce sterilising immunity [11]; therefore, modelling frameworks are needed to quantify the public health impact of a range of vaccination products. Previous studies have estimated the impact of maternal RSV vaccines using deterministic compartmental, cohort, and individual-based approaches and across different income settings [12–16]. One modelling study of maternal vaccine impact in England estimated that a seasonal immunisation programme could prevent 8.5 hospitalisations per 1000 vaccine courses administered [12], and a study based in Kilifi, Kenya, found that RSV infant infection could be reduced by up to 35% if maternal antibody protection duration is boosted to a total of 8 months [16]. Previously, we developed an age-structured, deterministic, compartmental model of RSV transmission, validated using RSV hospitalisation records for Western Australia, and estimated that a maternal vaccine could reduce infant RSV hospitalisations by up to 46% [17].

Household and cohort studies of RSV infection have suggested that households play a key role in RSV transmission and that household size and structure need to be considered when modelling RSV vaccination [18]. Although it is likely that older siblings are the primary source of infection within a household [19, 20], it is possible that in addition to protecting neonates from RSV infection, a maternal vaccine would provide extended protection to the mother, thus reducing household transmission [21].

The impact of household structures has been explored in several modelling studies of the predicted impact of RSV vaccine implementation in Kilifi, Kenya [13, 16, 19]. Brand et al. [13] developed a compartmental model with a household and community configuration, stratified into two age classes, and examined the relative impacts of vaccinating pregnant women and their household members. Poletti et al. [16] implemented a range of vaccine strategies, including a maternal vaccine, within an individual-based model that included households and schools. In a study of the impact of vaccinating older infants and children, Kinyanjui et al. [19] implemented a household structure within a deterministic age-stratified compartmental model of RSV transmission, parameterising contact patterns using household studies. However, we identified no modelling studies of maternal RSV vaccine impact that included households for a high-income setting. An RSV vaccine is expected to be implemented in countries across a range of income settings where demographic structure, life expectancy, household size, and contact patterns can vary substantially. Models developed for high-, middle- and low-income settings will therefore be needed, as estimates of future vaccine impact will likely vary depending on the setting.

In our study, we aimed to predict the household-level impact of a maternal RSV vaccine in a high-income country setting. We simulated RSV transmission within an individual-based framework, using an existing demographic model that is parameterised using Australian census and survey data [22, 23], linked to an epidemiological model of RSV. Our modelling framework allowed us to capture any herd immunity due to reduced household risk of infection conferred by immunisation, and to explicitly link the immune status of a mother to her newborn infant. We implemented a maternal vaccination strategy, delivered continuously throughout the year, and compared the infection incidence, which we expect to be a robust indicator of hospitalisations in very young infants, between vaccine- and non-vaccine scenarios.

## Methods

### Demographic model

We simulated population dynamics using a stochastic, individual-based model that accounted for births, deaths, couple formation, couple dissolution, and leaving home [22, 23]. We used Australian survival probabilities to calculate the number of births required in each of the preceding 100 years to achieve the 2017 Australian population age structure, scaled to a total population size of ~ 100,000 [24, 25]. Thereafter, while births and deaths occurred in the model, the population was non-growing and the age structure remained fixed at the 2017 distribution. Given the likely short-lived nature of RSV antibodies, as an extension to the published demographic model, we simulated pregnancy in this model in order to capture the timing of vaccination and prior infection in mothers [22, 23]. We explicitly accounted for the percentage of women in our population who will never have children, using ABS data on the number of children ever born by year of parental age [26].

Population mixing in our model occurs at both the household and community levels. Individuals in our modelled population were explicitly linked to their mothers and other members of their household. This structure allowed the implicit capture of within-household mixing, based on our assumption that an individual has contact with each member of their household every day. To account for mixing within the wider population, we employed age-based mixing parameterised using the mean daily number of contacts reported in POLYMOD, a large multi-country population survey [27]. As the POLYMOD numbers include all contacts, we reduced the reported age-specific mean daily number of contacts by the age-specific mean number of housemates in our modelled population [27]. Additional information on the demographic model and parameterisation of demographic processes is provided in Additional file 1 [24–26, 28–32].

### Epidemiological model

#### Model of infection and immunity

**Fig. 1:**
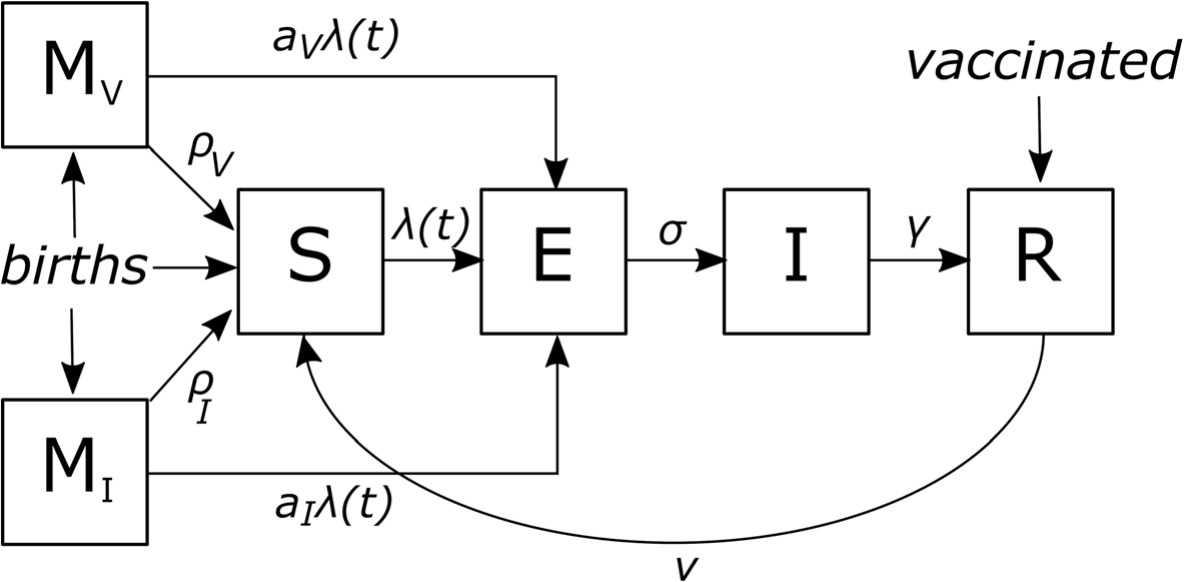
Schematic diagram of the epidemiological model. An infant is assigned one of three states at birth: a maternally protected state from the mother having been vaccinated (M_V_), a maternally protected state from the mother having experienced a recent infection (M_I_), or a fully susceptible state (S). If an infant would otherwise have both types of maternal protection, they are born into the M_V_ class. If exposed to infection (where exposure is scaled according to whether the infant has maternal protection), the infant moves to the exposed class (E), from which they progress to being infectious (I), and then to recovered (R). Vaccination of pregnant women can occur at any of the susceptible, exposed, infectious, or recovered states. Vaccinated individuals become fully protected from infection (R). Fully protected individuals lose protection over time and become fully susceptible once protection is completely lost. Parameter values are reported in Table 1

We combined an RSV transmission model with the individual-based demographic model and tracked the current state of infection or immunity for each simulated individual (Fig. 1). At birth, an infant is assigned one of three states: fully susceptible to infection (S), maternally protected due to their mother having been vaccinated during pregnancy (M_V_), or maternally protected due to their mother having recently been infected with RSV (M_I_), with infection possible from any of these states. Once infected, individuals become exposed (E), where they cannot transmit infection, before becoming infectious (I). Upon recovery, individuals become temporarily immune (R). Over time, an individual’s immunity wanes and they become fully susceptible to infection (S).

The force of infection acting on a fully susceptible individual (S) is a combination of their risks of household and community acquisition. These risks are derived from an individual’s age-specific number of community contacts, the number and ages of infectious individuals in the community, and the number and ages of infectious individuals in their household. When calculating the force of infection, individuals older than 10 years are assumed to be less infectious than those aged 10 and younger. Maternally protected individuals (M_V_ and M_I_) are partially protected from RSV infection, with the force of infection acting on these individuals reduced by factors *a*_*V*_ and *a*_*I*_, respectively. To replicate seasonal patterns in RSV incidence observed in temperate settings, seasonal forcing was included, using the value determined from a previous compartmental model calibrated to Australian data [17].

#### Vaccination

Upon becoming pregnant in our model, a woman is assigned a date on which she will be considered for vaccination, with this date drawn from a uniform distribution ranging from 6 weeks to 3 months before she is scheduled to give birth. On this date, the woman becomes vaccinated with a probability equal to the desired vaccination coverage for the simulation and, if vaccinated, becomes temporarily immune (R) for a duration drawn from a Gamma distribution (mean 230 days, shape parameter 3). Considering reported coverage of the maternal influenza and pertussis vaccines in Australia, we assumed a baseline coverage of 70% and tested coverage values in the range 30–100% [33–37].

#### Infant immunity

It is known that pregnant women have pre-existing RSV-specific antibodies due to prior infection with RSV and that these antibodies are transferred across the placenta to provide protection to the newborn infant. An RSV vaccine delivered antenatally during the third trimester is expected to boost the level of RSV-specific antibody that is available for transfer [11]. We therefore aimed to mimic this immunological relationship by explicitly linking infant immune status with that of their mother. Vaccine-derived infant immunity was assumed to be relative to the mother’s remaining duration of immunity at birth and was modelled as follows. At the time of vaccination, each pregnant woman was assigned a duration of immunity drawn from a Gamma distribution (Table 1). When her infant was born, the remaining immune time (in days) was multiplied by the scaling factor $1/\left[{\rho}_V\left(\frac{1}{\nu_V}-64\right)\right]$, where 1/*ρ*_*V*_ is the mean duration of infant vaccine-derived protection from birth, and 1/*ν*_*V*_ is the mean duration of immunity following vaccination. Therefore, the duration of infant immunity was such that a mother who was vaccinated in the middle of the vaccination window (i.e., 64 days before birth) and was assigned the mean duration of immunity (230 days in the baseline scenario) would pass 90 days of protection to her infant. The 90 day duration was selected based on published clinical trial endpoints and the WHO minimum acceptable duration [3, 9]. The duration of immunity for infants born to vaccinated mothers therefore depended on both the timing of vaccination and the assigned duration of immunity for the mother.

**Table 1:** Epidemiological parameters

Notation	Description	Selection method	Baseline value (alternative values)	Reference
	Vaccine coverage	Fixed	70% (30%, 50%, 100%)	[33–37]
1/*ρ*_*V*_	Mean duration of protection after birth (from vaccination) in days	Calculated based on number of days of mother’s immunity remaining at birth	90 (182, 230) days. See text for explanation.	[9, 10]
1/*ρ*_*I*_	Mean duration of protection after birth (from infection) in days	Calculated based on number of days of mother’s immunity remaining at birth	90 (182, 230) days. See text for explanation.	
*a*_*V*_	Reduced susceptibility to infection in infant from mother having been vaccinated	Fixed + sensitivity analysis	0.4 (0.2, 0.6)	
*a*_*I*_	Reduced susceptibility to infection in infant from mother having been infected	Fixed + sensitivity analysis	0.4 (0.2, 0.6)	[17]
1/*σ*	Latent period (days)	Gamma distribution, shape parameter 3	4	[38, 39]
1/*γ*	Infectious period (days)	Gamma distribution, shape parameter 3	9	[39, 40]
1/*ν*_*I*_	Duration of immunity following infection	Gamma distribution, shape parameter 3	Mean 230 days (182, 364)	[41]
1/*ν*_*V*_	Duration of immunity following vaccination	Gamma distribution, shape parameter 3	Mean 230 days (182, 364)	
*ω*	Reduced infectiousness in individuals aged 10 years and over	Calibration	0.2	See text
*q*	Community transmission coefficient	Calibration	0.015	See text
*q*_*h*_	Household transmission coefficient	Calibration	2.4	See text
*b*_*1*_	Amplitude of seasonal forcing	Fixed	0.397	[17]

For infants deriving protection from their mother’s recent RSV infection, the remaining immune time at birth was similarly scaled by $\left(1/{\rho}_I\right)/\left(1/{\nu}_I\right)$, where 1/*ρ*_*I*_ is the mean duration of infant infection-derived protection from birth, and 1/*ν*_*I*_ is the mean duration of immunity following infection. Therefore, the duration of infant immunity was calculated such that a mother who was infected, and recovered on the day of her infant’s birth and was assigned the mean duration of immunity (230 days in the baseline scenario) would pass 90 days of protection to her infant, and a mother infected, and recovered, prior to her infant’s birth would pass on a corresponding proportional reduced length of immunity. The duration of immunity for infants born to recently infected mothers therefore depended on both the timing of the mother’s infection and the assigned duration of immunity.

#### Model calibration

To calibrate the model, we identified several key characteristics of RSV epidemiology to which we simultaneously compared simulated outputs generated across a range of combinations of the community and household transmission coefficients (*q* and *q*_*h*_) and the reduced infectiousness parameter (*ω*) (Table 1). In temperate climates, including in much of the Australian setting, RSV incidence is typically observed as marked annual or biennial winter peaks [42]. We therefore retained parameter value combinations for which the model produced annual peaks or biennial peaks in RSV incidence and discarded those that produced biannual or endemic dynamics. In a Western Australian cohort study, it was estimated that 45% of the RSV detections in infants aged less than 1 year were attributable to an older sibling [20]. We calculated the proportion of infections in infants under 1 year old that were caused by household members younger than 18 years and discarded parameter combinations that produced proportions outside the range 35–50%. It is widely estimated that almost all children are infected by RSV within their first 2 years of life and that approximately two-thirds are infected before the age of 1 year [43, 44]. We therefore extracted the number of RSV infections in children younger than 1 year of age and retained parameter combinations that produced annual incidence between 60,000 and 70,000 per 100,000 in this age group.

Six combinations of the community and household transmission coefficients (*q* and *q*_*h*_) and the reduced infectiousness parameter (*ω*) simultaneously met the three filtering conditions. All of these had *q* = 0.015 and *ω* = 0.2. The value for *q*_*h*_ was selected as 2.4, as this value produced incidence closest to the desired outcome of two-thirds of infants being infected in the first year of life. Parameter sweep values and the results of the calibration process are presented in Additional file 1: Figure S2.

#### Simulations

Prior to each simulation, our 2017 model population was seeded with five infectious individuals and was run for a 10-year burn-in period to reach endemic disease equilibrium. From this starting point, baseline results were obtained by running the model for a further 10-year period with no vaccination.

Four maternal vaccination scenarios were explored, with effective vaccination coverage of 30%, 50%, 70%, and 100%. In each scenario, the model was run for 5 years without vaccination (pre-vaccination period), followed by 5 years with vaccination (post-vaccination period). For each model simulation run, the percentage change in average annual incidence between the post-vaccination and pre-vaccination periods was calculated, discarding the first year of vaccination as a burn-in. For each model simulation run, we calculated incidence rate ratios (IRRs) in the post-vaccination period, comparing average annual incidence in infants with vaccinated mothers to infants with non-vaccinated mothers. Twenty-five simulations were run for the baseline and each of the four vaccination scenarios.

#### Sensitivity analysis

We analysed model sensitivity to assumptions about the duration and strength of both natural- and vaccine-induced immunity. Keeping all other parameters at their baseline values, we simultaneously varied the duration of infection- and vaccine-induced immunity over all combinations of values in Table 1. Similarly, we simultaneously varied the reduced susceptibility to infection derived from both infection and vaccine immunity, again over all combinations of values in Table 1. Additionally, we varied the mean duration of infant immunity over the values in Table 1 while keeping all other parameters at baseline values. Twenty-five model simulations were run for each parameter combination. We report the distribution of the percentage change in average annual incidence by age.

The model was run in Python programming language version 3.5 [45], and the results were analysed using R version 3.4.4 [46].

## Results

### Effect of maternal vaccination

#### Infant immunity at birth—percent immune and median duration

**Fig. 2:**
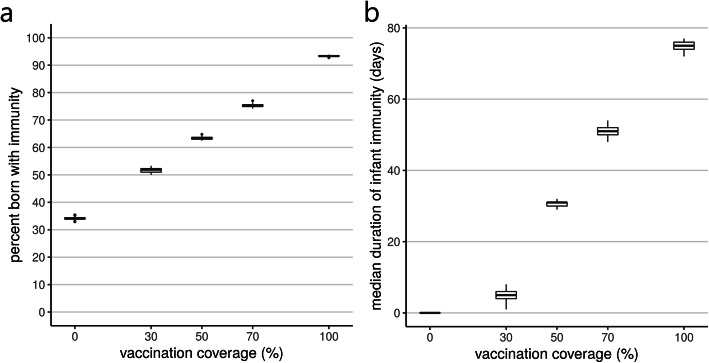
**a** Median percentage of infants born with any immunity and **b** median duration of infant immunity. For each effective vaccination coverage, the box shows the distribution (median, IQR) over 25 simulations

According to the model, without maternal vaccination in place, 34% (interquartile range (IQR) 34–34) of infants would be born with some immunity to RSV, resulting in all baseline simulations producing a median duration of immunity of 0 days. The percent of infants born with any immunity increased linearly with vaccination coverage (Fig. 2a), reaching a median of 93% (IQR 93–93) when all mothers received effective vaccination. The median duration of immunity at 30% coverage was 5 days (IQR 4–6), rising to 75 days (IQR 74–76) when all mothers were vaccinated (Fig. 2b).

#### Percent change in annual incidence

**Fig. 3:**
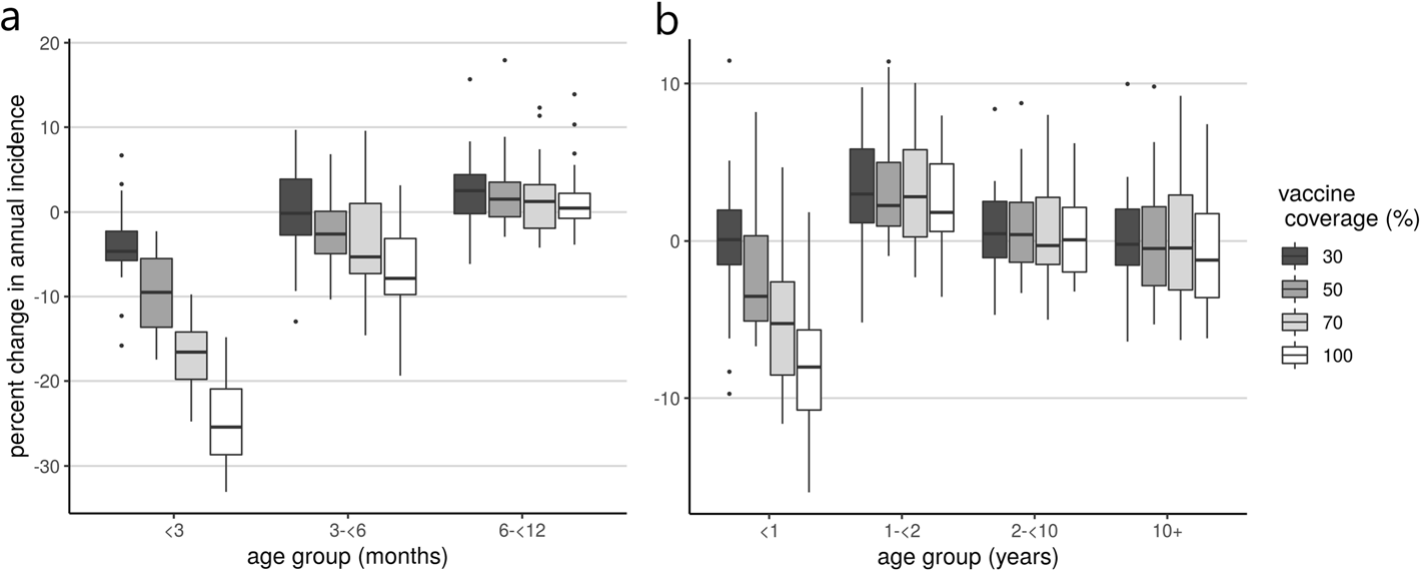
The percent change in annual infection incidence, comparing post-vaccination and pre-vaccination periods for **a** infants under 1 year of age and **b** the whole population

Under the best-case scenario of 100% vaccination coverage, the greatest benefit of vaccination was observed in infants younger than 3 months, with population incidence of infection reduced by 25.5% (IQR 20.9–28.7) (Fig. 3a). Based on coverage levels for other maternal vaccines, we expect 70% coverage is the most plausible of the scenarios we simulated. With 70% coverage, infants younger than 3 months still experienced the greatest benefit of vaccination, with the population incidence of infection reducing by 16.6% (IQR 14.2–19.8). A modest reduction of 5.3% was seen in infants aged 3 to 6 months, with the IQR ranging from a decrease of 7.3% to an increase of 1.0%. Median incidence of infection was reduced in children younger than 6 months even at very low levels of vaccine coverage. The infection incidence in most older age groups remained similar to pre-vaccination levels, except for the 1–2 years age group, where an increase of 2.8% (IQR 0.3–5.8) was observed (Fig. 3b). Age-specific annual incidence of infection post-vaccination is provided in Additional file 1: Figure S3.

#### Effect of household size on the impact of maternal vaccination

The impact of maternal vaccination reduced as household size increased. The largest reduction was observed for infants living in a household of two (one adult and one infant), with an approximately 30% reduction in the mean annual infant cases, although it is important to note that there are few of these households and the majority of cases occur in households of size three and four (Additional file 1: Figure S4). For families with five and six members, the mean annual infant cases increased.

#### Effect of maternal vaccination on infants born to vaccinated mothers

Over the first 6 months of life, the IRR between infants born to unvaccinated mothers and those born to vaccinated mothers was 1.26 (IQR 1.23–1.30) at 70% vaccination coverage and was relatively invariant to changes in vaccination coverage (Additional file 1: Table S1).

### Sensitivity analysis

#### Susceptibility to infection

**Fig. 4:**
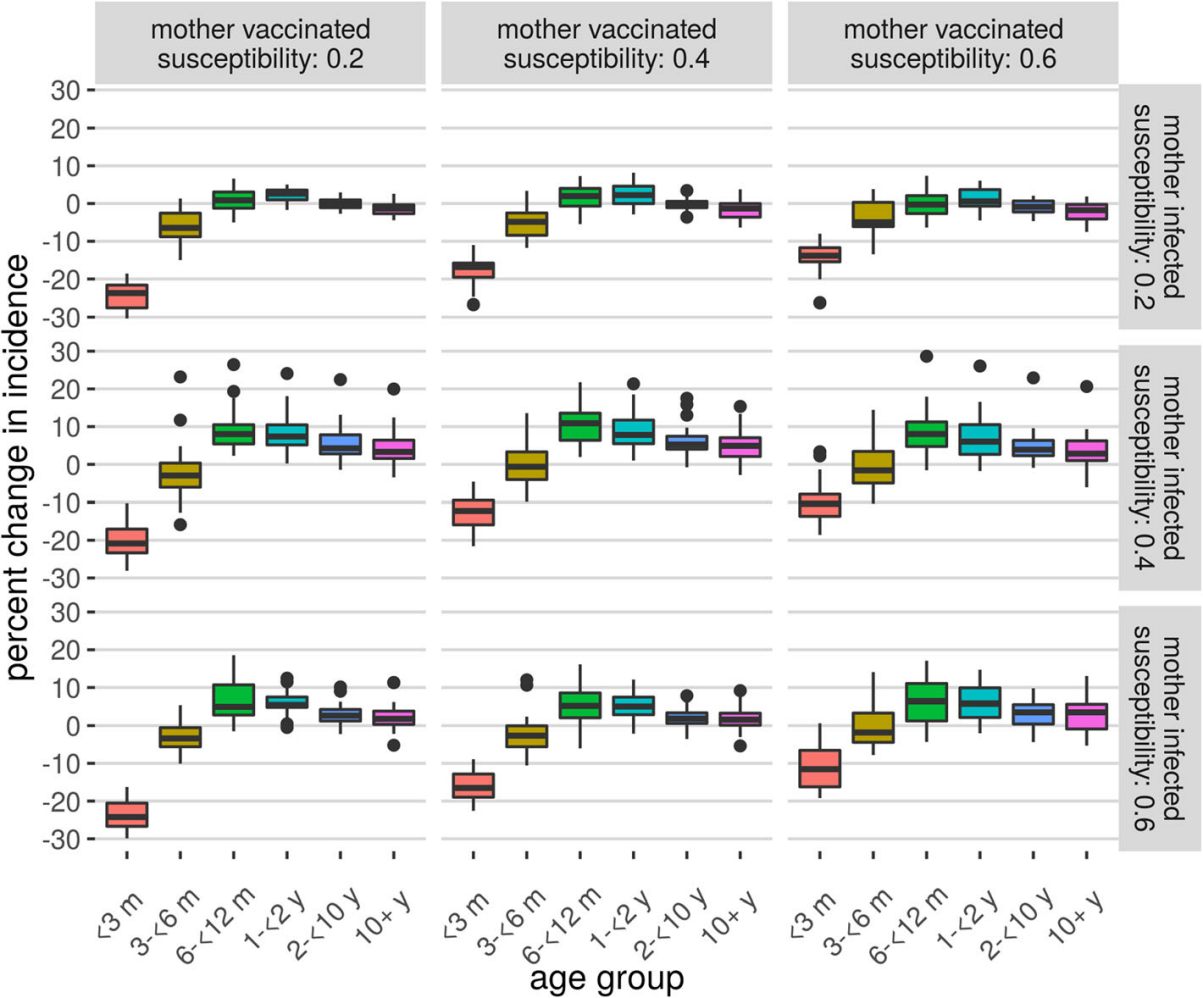
Sensitivity analysis for the susceptibility parameters. Each row represents a different value of the susceptibility of infants born to infected mothers, and each column the same for infants born to vaccinated mothers, with results showing the percentage reduction in incidence. The baseline assumption used susceptibility multipliers of 0.4 for infants born to vaccinated or infected mothers, compared to completely susceptible infants. All other parameters were fixed at their baseline assumption

Our baseline assumption was that infants born to vaccinated or infected mothers experienced a force of infection 0.4 times that of a completely susceptible infant. The percentage reduction in incidence for the youngest age group (less than 3 months of age) was sensitive to the extent that the vaccine prevented infection, with the effect of vaccination roughly doubling when we changed our susceptibility multiplier from 0.6 to 0.2 (Fig. 4). This sensitivity was not observed in other age groups. The reduction in incidence post-vaccination was reasonably stable across different values of the susceptibility multiplier for infants born to infected mothers. Similar trends were observed in the IRRs between infants born to unvaccinated mothers and those born to vaccinated mothers over the first 6 months of life (Additional file 1: Table S2).

#### Duration of immunity

The percent reduction in incidence for infants under 3 months of age was relatively stable across different durations of vaccine-induced immunity and showed minor variability across different durations of infection-induced immunity (Additional file 1: Figure S5). The percent reduction in incidence in this age group ranged from 9.4% (IQR 6.0–17.0) when both infection and vaccination provided 182 days of immunity to 18.7% (IQR 12.2–24.6) when both provided 364 days of immunity.

With vaccination coverage of 70%, over the first 6 months of life, the IRR between infants born to unvaccinated mothers and those born to vaccinated mothers increased slightly as the duration of infant immunity was increased (IRR 1.26 (IQR 1.23–1.30) at 90 days vs IRR 1.33 (IQR 1.31–1.35) at 230 days) (Additional file 1: Table S3).

## Discussion

Development of RSV vaccines is a priority for the WHO to reduce the burden of disease in young infants. Phase 3 clinical trial results for maternal vaccination, designed to protect infants through antenatal transfer of antibodies, have shown that such a vaccine is likely to be imperfect [10, 11]. Using an individual-based modelling approach that incorporated household structure and vaccination during the third trimester of pregnancy, we have shown that maternal vaccination is likely to increase the percentage of infants born with any immunity to RSV, from 34% without vaccination to 75% when 70% coverage is achieved and 93% with 100% vaccination uptake. The median duration of immunity at birth rises from 0 days when no maternal vaccination is in place to 51 days when 70% coverage is achieved. The reduction in incidence in infants in the first 3 months of life is likely to be around 17% falling to 5% for the 3–6-month age group. The benefit to individual infants is somewhat greater, with infants born to unvaccinated mothers experiencing 26% higher incidence levels than those born to vaccinated mothers. The degree of protection maternal vaccination provided to the very youngest infants (less than 3 months) was a key determinant of both the population level and individual level reduction in incidence, but this sensitivity did not extend to older age groups. The percentage reduction in incidence was relatively invariant to changes in the duration of protection provided by maternal vaccination, while some dependence on our assumptions about the duration of protection following infection was observed. We also found that over the first 6 months, the relative RSV incidence between infants born to unvaccinated mothers and those born to vaccinated mothers was consistent across different levels of vaccination coverage, suggesting little to no herd immunity impact resulting from vaccination.

RSV is a seasonal disease, with the season generally spanning three to four winter months in the temperate regions of Australia. Other temperate regions typically experience annual RSV epidemics over a period of 3 to 6 months, and seasonal patterns differ in the tropics [47]. This seasonality likely plays an important role in the modelled impact of a maternal RSV vaccination program, as in the off-season, there is little to no benefit provided by maternal vaccination as the risk of infection is so low. The seasonality and duration of the season also explain why, within limits, the duration of protection provided by a maternal vaccine has a relatively minor influence on the incidence reduction. For many infants, preventing an infection in the first 3 months of life is likely to push their first infection into the next year. Infants born towards the end of the RSV season were already unlikely to be infected before they reach the age of 6 months. Therefore, while year-round administration of a vaccine is likely to be the most operationally feasible and equitable approach and is the strategy currently recommended for maternal influenza immunisation in Australia, seasonality is predicted to be important in terms of vaccine impact, and it is possible that a seasonal maternal RSV vaccine schedule may be considered in other jurisdictions.

It is understood that households are important drivers of RSV transmission [18]. We observed the largest reduction in numbers of infant infections in households of three people, and the largest percentage reduction in infant infections in households of two (although there are relatively few households of this size containing infants). For larger households, the impact of maternal vaccination decreased, and for households with five and six members, the mean annual number of infant infections was slightly higher after the introduction of a vaccine. This increase may be the result of the increased incidence observed in older children after the introduction of vaccination, with infants born into larger households more likely to have siblings in the affected age groups.

Even when all mothers in our model were immunised (100% coverage scenario), not all infants were born with protection against infection, due to a mother’s immunity having waned prior to delivery. We based the duration of protection received by an infant at birth on the duration of immunity their mother had at the same time, using the mother’s remaining duration as a proxy for the level of antibodies that could be passed to an infant via placental transfer. The duration of protection remaining for a mother depended on two factors, namely when the mother was vaccinated (chosen randomly between 6 weeks and 3 months before birth) and the duration of her own protection (chosen randomly from a Gamma distribution with mean 230 days). The interaction of these two factors resulted in the immunity for some mothers having completely waned before their baby was born. More data on the antibody levels in cord blood and infants is needed to confirm the extent to which this does in fact occur.

There are few published mathematical and computational modelling-based estimates of maternal RSV vaccine impact, and the available studies differ in terms of how results are presented, making it difficult to make direct comparisons. Brand et al. [13] incorporated household configuration and communities into an SIR model of RSV transmission, with two age classes and parameterised for the setting of Kilifi, Kenya. They estimated that with 50% coverage and 90 days of protection, a maternal vaccine could reduce hospitalisations by 21.6% and total infections by 0.13%, although this was relative to the entire population across all age groups. At 50% coverage, we estimated a reduction in RSV infections of 0.5% (IQR − 2.2–2.8) in individuals aged 10 years and older. Pan-Ngum et al. [15], using two distinct compartmental age-structured models, estimated that a maternal vaccine could reduce hospitalisations by 7–15%, depending on the model and vaccine characteristics. A decision-tree model for the USA setting estimated that a maternal vaccine strategy with 56% uptake, 80% efficacy, and 90 days of protection, combined with the current recommended immunoprophylaxis therapy palivizumab for high-risk infants, would prevent 14% of RSV-associated lower respiratory tract infections in infants younger than 12 months presenting to the outpatient clinic, and 25% of RSV hospitalisations, relative to palivizumab alone [48].

Our study has several strengths. First, we captured the immunological mechanism of transfer of protective RSV-specific antibodies by explicitly relating the immune status of the mother to that of her infant. This allowed us to incorporate the seasonality of RSV infection, and the seasonality of natural antibody transfer when derived from recovery following prior infection. Seasonal protection in neonates as a result of prior infection has not generally been incorporated into other RSV transmission models [12]. Second, our study is one of the first to incorporate households and demography into a model of maternal RSV vaccination in a high-income setting. Predicting the impact of RSV vaccination across a range of income settings will be crucial for allowing decisions to be made about vaccine policy, given the substantial global health burden of RSV, and considering that an RSV vaccine is targeted for near-concurrent introducing in high-, middle-, and low-income settings [5]. Third, RSV is a non-notifiable disease in Australia, and therefore, data typically available, such as for hospitalisations, represent the more severe end of the disease spectrum. This limits the available data to younger age groups, making it difficult to ascertain whether modelled incidence in older age groups represents reality. Rather than fitting the model to hospitalisation data, as we did for our previous compartmental model [17], we instead ran parameter sweeps for parameters directly related to the force of infection. We then retained the parameter combination that most closely replicated three key characteristics of RSV that we expect would be robust across different disease manifestations, namely the presence of a single annual peak, incidence rates consistent with around two-thirds of infants experiencing infection before their first birthday, and a high proportion of infections in infants being caused by an infected sibling.

A limitation of our study is that we focussed on the impact of infections, rather than symptomatic disease and hospitalisations. Even though we modelled infection, we anticipate that the reduction in infection in the model would translate to a reduction in disease. However, this relationship may not hold if the action of the vaccine were to prevent pathogenesis, rather than infection. Further, in our model, we captured vaccine protectiveness by reducing susceptibility to infection by a scaling factor, and vaccine-derived protection was not explicitly differentiated from protection following recovery from natural RSV infection. Our measure of reduced susceptibility therefore does not directly align with trial efficacy and limits the ability to directly compare model parameters with clinical trial endpoints, although we expect that they would be correlated.

In this study, we focussed on the impact on RSV incidence, stratified by age group, as our main outcome measure. However, given the hierarchy of efficacy with severity of disease [10], the impact is anticipated to be larger for outcomes of severe disease, hospitalisations or mortality, and this will be an avenue for future work. It is also expected that a vaccine would have particular benefit for at-risk groups, including infants with comorbidities, although children born very prematurely may not benefit from maternal vaccination due to the timing of administration in the third trimester of pregnancy and the limited transfer of maternal antibodies before that timepoint [49]. Quantifying the impact of RSV vaccination for children at highest risk of severe disease is an area of future research.

## Conclusions

In conclusion, we have validated an individual-based model that captures RSV transmission dynamics due to household structure, using robust key criteria. Our simulations show that immunisation of pregnant women could be an effective strategy to reduce the burden of RSV in young children, particularly in infants younger than 3 months of age, although impact in older age groups may be small. While we focussed on a high-income setting, our modelling framework could readily be adapted to predict RSV vaccine impact in other income settings by modifying the underlying population demography. In addition, our model is flexible enough to be used for estimating impact of other RSV vaccine products, such as a childhood or infant vaccine, and can be updated to incorporate more data from RSV vaccine trials as pharmaceutical development progresses.

## Supplementary information

**Additional file 1.** Content: additional methods and results.

## Data Availability

The demographic and epidemiological model code, and associated input data files, are freely available to download at https://bitbucket.org/TrishC/rsv-ibm/src/master/. All other data generated or analysed during this study are included in this published article and its supplementary information files.

## Abbreviations

RSV: Respiratory syncytial virus
WHO: World Health Organization
LRTI: Lower respiratory tract infection
IRR: Incidence rate ratio
IQR: Interquartile range
