# Supplementary material for: Modelling the household-level impact of a maternal respiratory syncytial virus (RSV) vaccine in a high-income setting
Source: BMC Med. 2020 Nov 12;18:319. doi: 10.1186/s12916-020-01783-8 (PMC7661211; doi:10.1186/s12916-020-01783-8)
Supplement: Supplementary file 1 — Additional file 1. Content: additional methods and results. [file 12916_2020_1783_MOESM1_ESM.pdf]

Supplementary Material

Modelling the household-level impact of a maternal respiratory syncytial virus (RSV) vaccine in a high-income setting

Patricia Therese Campbell, Nicholas Geard, Alexandra B. Hogan

Outline of the demographic model

We use an individual based model, in which each individual is characterised by age, sex, household of residence, and family links. Over time, individuals are born, age, form and dissolve couples and household units, and die, with probabilities determined by their sex, current age and life stage. The population demography is updated at each time step of the simulation (in the simulations reported here, at daily intervals) according to the following procedure:

1. The age of each individual is incremented by one day.
2. For each individual i, one of the following events may occur:
   (a) **Death:** with a probability based on i's age and sex, i dies and is removed from the population. An individual j is chosen to be the mother of a replacement individual as follows:
      i. The target age of the mother is determined on the basis of age-specific fertility rates.
      ii. A set of candidate mothers is determined on the basis of age, eligibility to give birth and household status (for simplicity, individuals are not eligible to give birth while living with their own parents).
      iii. j is selected at random from the pool of candidate mothers.

   If the death of i results in a household containing only children, these individuals are reallocated as follows:
      i. Any children aged 18 or older form new single-person households.
      ii. Any children aged less than 18 are randomly allocated (fostered) to other households containing at least one child.

(b) **Couple formation:** if i is currently single, with a probability based on i's age, i forms a couple with an individual j, chosen as follows:

   i. A set of candidate partners is determined on the basis of age, sex, and not currently being a member of a couple.

   ii. j is selected at random from the pool of candidate partners.

   The households of i and j are merged (along with any children currently residing with them) or, if both previously lived with their parents, a new household of size two is created.

(c) **Leaving home:** if i is currently living with their parents, with a probability based on i's age, i leaves their parents' household and forms a new household of size one.

(d) **Couple separation:** if i is currently in a couple, with a probability based on i's age, i separates from that couple and forms a new household; for simplicity, we assume that any children residing with the couple when they separate join the mother's household.

Population model parameters and data sources

Mortality: Age-specific mortality rates for Australia were sourced from the Australian Bureau of Statistics [28]. For convenience, we assume that no individual survives beyond 100 years, and the probability of death at 100 years was fixed at 1.0.

Fertility: Age-specific fertility rates for Australia were sourced from the Australian Bureau of Statistics [24]. These rates were not used directly to generate births in our model, but rather used to estimate relative probabilities of births being attributable to mothers of a particular age. When a birth event was triggered, these relative probabilities were used to ascertain the age of the mother, and hence the subset of the female population eligible to be randomly chosen as the mother.

Women without children: A subset of women in Australia never have children. We assigned a flag to 13% of females when they were born, so that they were never selected as a candidate mother. We based this percentage on the proportion of women in Australia who have no children by the time they reach the age of 55 years, sourced from the Australian Bureau of Statistics dataset on the number of children ever born by age and sex of parent [26].

Couple formation and separation, leaving home: Probabilities were estimated on the basis of data on the Australian population reported by the Australian Institute of Family studies [29] and the Household Income and Labour Dynamics in Australia Survey [30]. This estimation combined data reported both on rates of marriage and divorce with data on rates of de-facto relationships, as the primary focus of our model was the dynamics of household units, rather than the status of relationships. We assume that individuals become eligible to leave their parents' household, either independently or as a member of a couple, at 18 years. As a consequence, individuals also

become eligible to separate from a couple at 18 years. We assume that individuals cease being eligible to form or separate from couples at 60 years.

The primary aims of the demographic model were to capture a reasonable approximation of the size and composition of households in the Australian population in 2017 and to execute in a computationally efficient fashion. It is not feasible that the model accurately capture all the demographic complexity of a real population and, as described above, several simplifying assumptions have been made in the name of model parsimony. For example, our model currently simulates dynamics of households containing one or two adults/parents (of opposite sex) and zero or more children (as defined by their familial relationship to the parents in the household; they may themselves be adults who are yet to leave home). Clearly, this does not exhaust the potential range of household types observed in real populations. Furthermore, our model does not include immigration, which may introduce individuals of a range of ages.

Generating the starting population

We generated a population of 100,000 people with an age distribution corresponding to that of Australia in 2017, obtained from the Australian Bureau of Statistics [25]. To do this, we first fit a Siler survival probability function to Australian survival probabilities obtained from the World Health Organization Life Tables [31]. We then used this function to calculate the number of births required in each of the preceding 100 years to produce the target age distribution, scaled to a population size of 100,000.

Once the population corresponding to 2017 had been generated, demographic rates were assumed to remain stable over the period covered by the scenarios compared in the paper, on the basis that fertility rates — a key demographic driver of epidemiological dynamics — have remained relatively stable (at just under 2 births per woman) over recent decades in Australia.

A comparison of model demographics to Australian data is shown in Supplementary Figure S1. The age distribution extracted from the model at the end of the demographic burn in period is compared with the 2017 Australian age distribution [25]. The household size distribution extracted from the model at the end of the demographic burn in period is compared to the 2016 Australian household size distribution [32].

Supplementary Figure 1

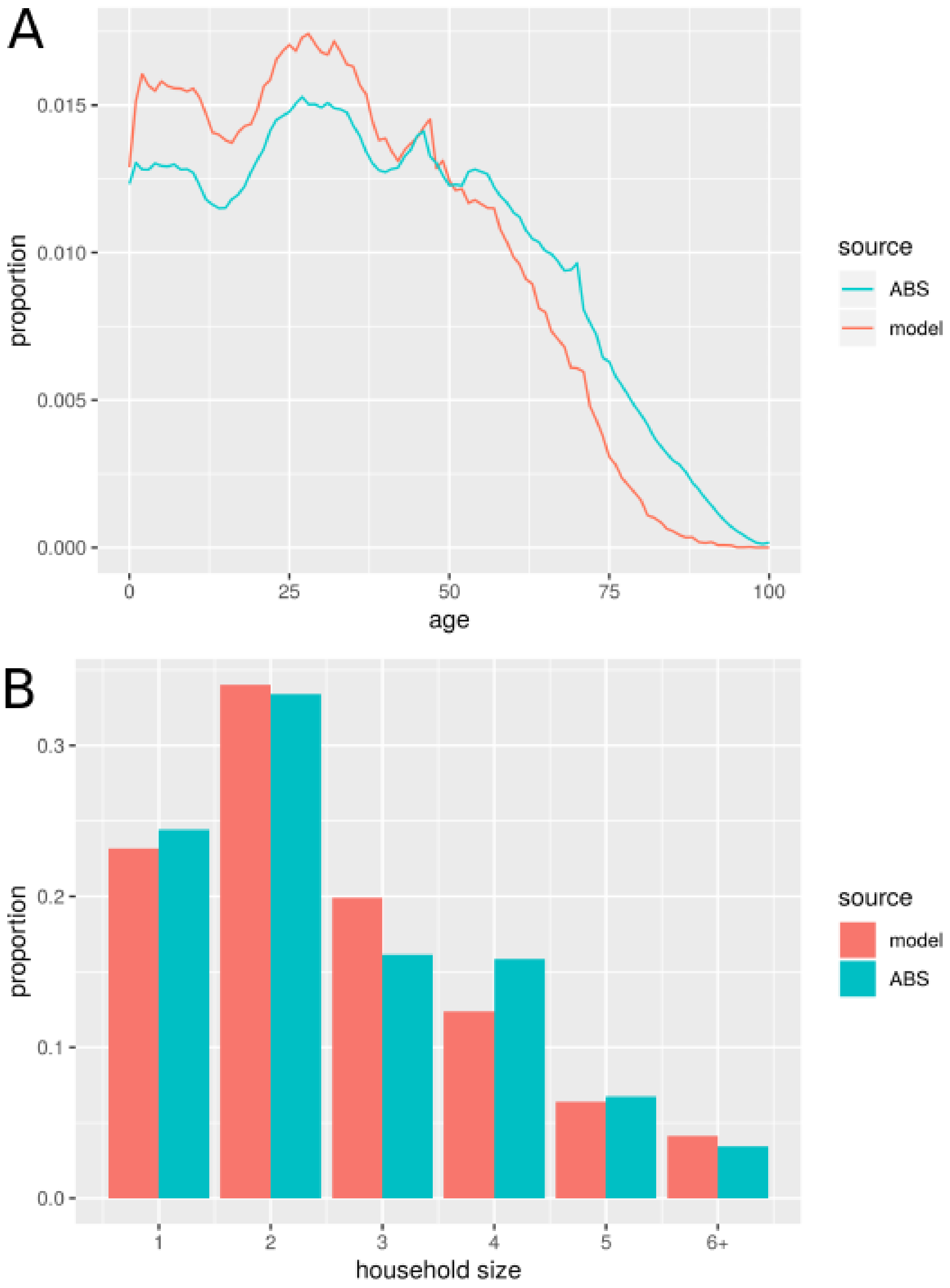

Figure S1: Comparison of model demographics with Australian data for A) Age distribution; and B) household size distribution.

Supplementary Figure 2

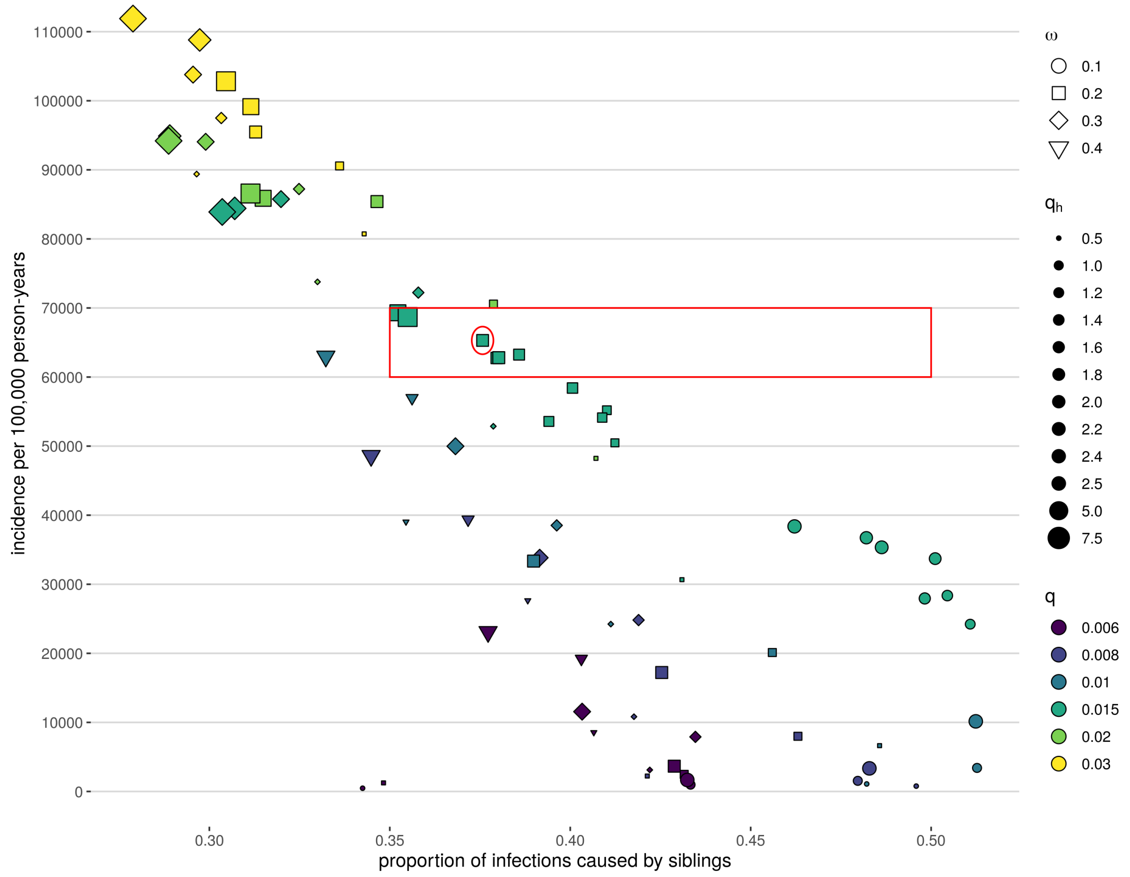

Figure S2: Calibration results. Values used for the parameter sweeps were $q \in 0.006, 0.008, 0.01, 0.015, 0.02, 0.03$ (plotted by colour), $q_h \in 0.5, 1.0, 1.2, 1.4, 1.6, 1.8, 2.0, 2.2, 2.4, 2.5, 5.0, 7.5$ (plotted by size) and $\omega \in 0.1, 0.2, 0.3, 0.4$ (plotted by shape). The desired ranges for the model outputs, the proportion of infections caused by siblings (0.35–0.50) and the infant incidence in the first year of life (60,000–70,000), are framed by the red box. The selected parameter combination used for model simulations was $q = 0.015, q_h = 2.4$ and $\omega = 0.2$, circled in red. We observed a negative correlation between infant incidence and the proportion of infections caused by siblings. Intuitively this makes sense: higher levels of incidence mean more infection is circulating in the community and thus it is more likely that an individual will be infected by a source outside of the household.

Supplementary Figure 3

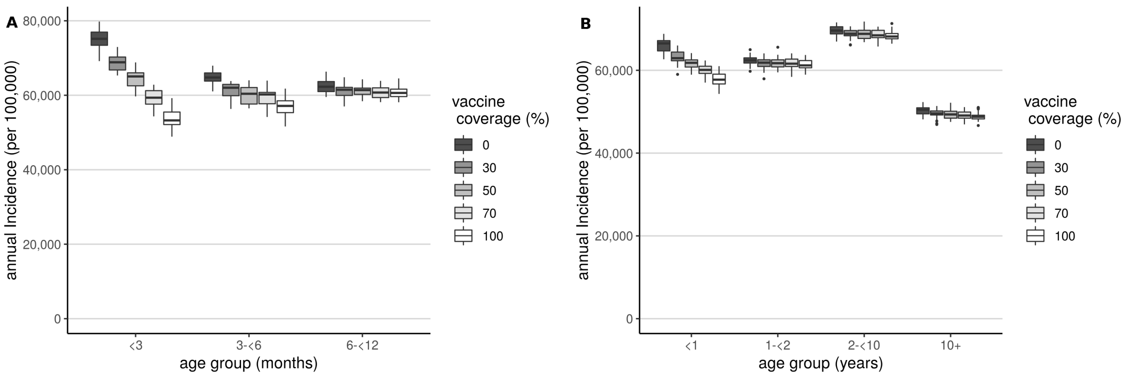

Figure S3: Annual incidence of infection post-vaccination across a range of effective vaccination coverage in A) infants under one year of age; and B) the whole population.

Supplementary Figure 4

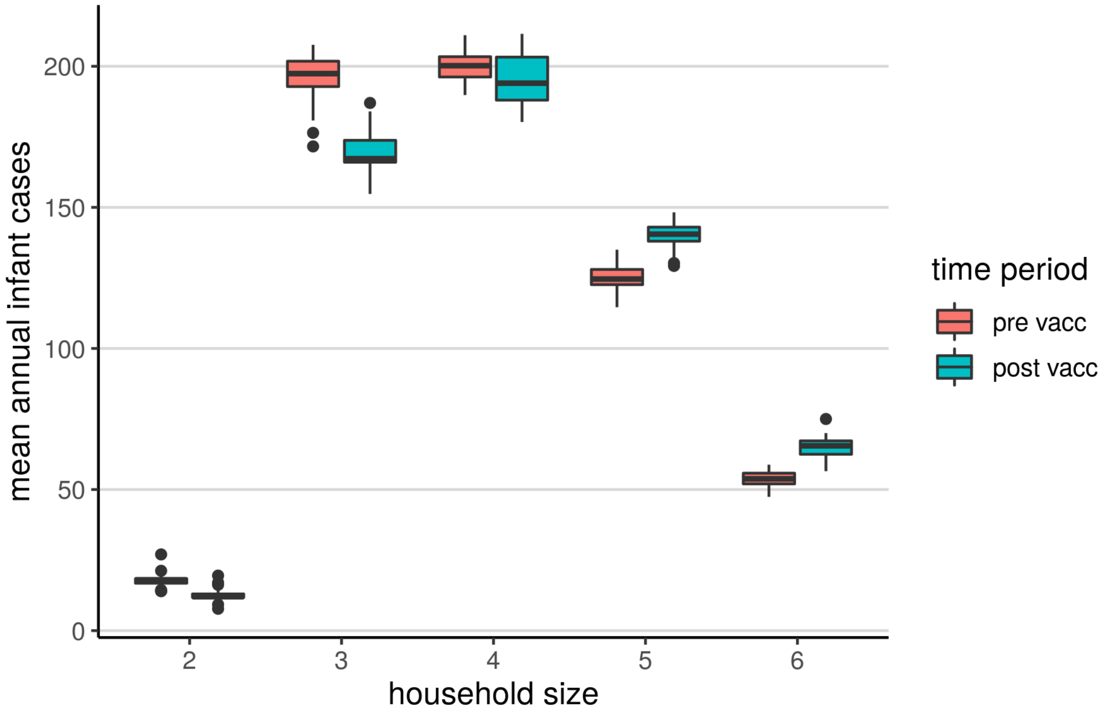

Figure S4: Mean annual infant cases in the pre-vaccination and post-vaccination period stratified by household size. The population living in households of each size remained relatively stable across the time frame covered by these simulations. Simulations were run with the baseline parameter set and maternal vaccination coverage set to 70%.

Supplementary Figure 5

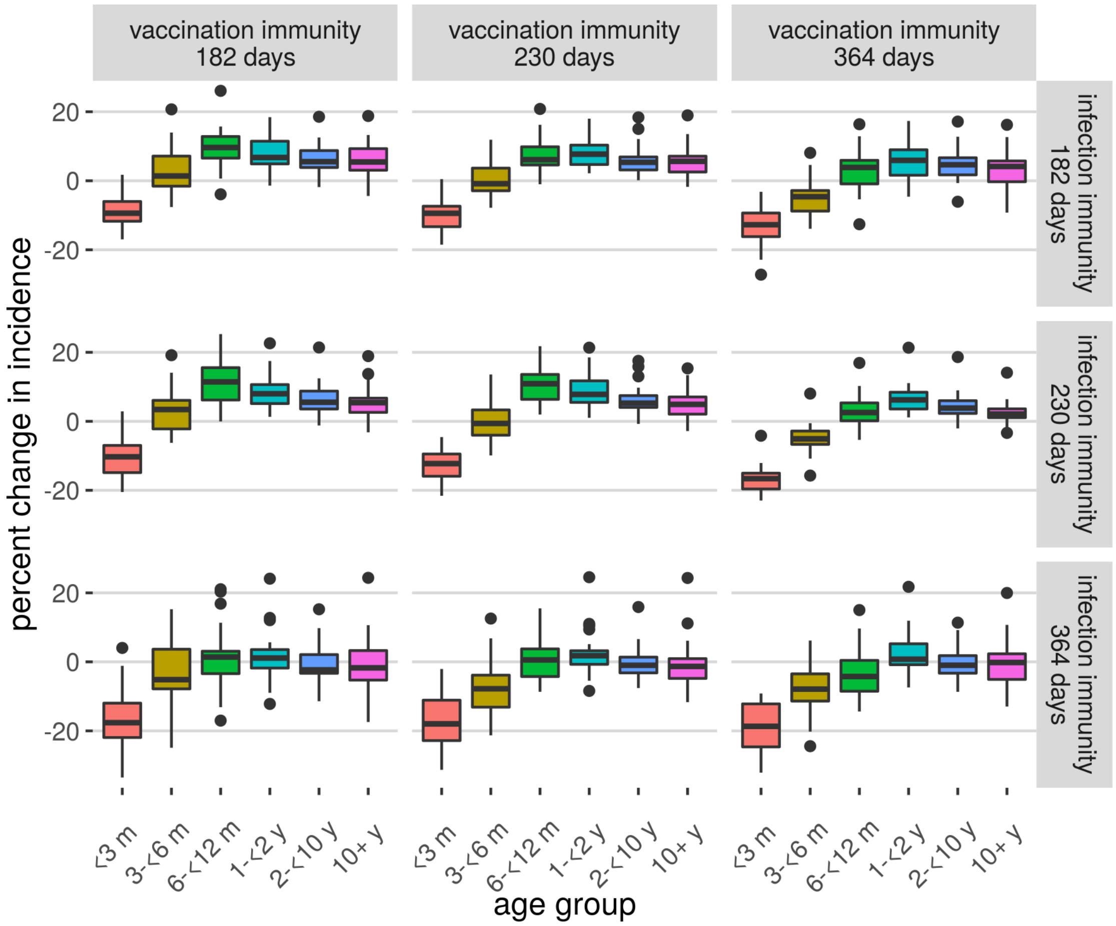

Figure S5: Percent reduction in incidence for all age groups across different durations of vaccine- and infection-induced immunity

Supplementary Table 1

Table S1: IRRs comparing incidence over the first six months of life for infants born to unvaccinated mothers relative to incidence for infants born to vaccinated mothers.

Vaccination coverage (%)	Median IRR	IQR
30	1.27	1.22–1.30
50	1.25	1.21–1.29
70	1.26	1.23–1.30

Supplementary Table 2

Table S2: IRRs comparing incidence over the first 6 months of life for infants born to unvaccinated mothers relative to incidence for infants born to vaccinated mothers, over different combinations of susceptibility multipliers.

Susceptibility (vaccine)	Susceptibility (infection)	Median IRR	IQR
0.2	0.2	1.34	1.32–1.39
0.4	0.2	1.25	1.22–1.29
0.6	0.2	1.23	1.19–1.25
0.2	0.4	1.39	1.35–1.39
0.4	0.4	1.25	1.22–1.28
0.6	0.4	1.25	1.20–1.28
0.2	0.6	1.39	1.33–1.41
0.4	0.6	1.29	1.26–1.32
0.6	0.6	1.23	1.18–1.35

Supplementary Table 3

Table S3: IRRs comparing incidence over the first six months of life for infants born to unvaccinated mothers relative to incidence for infants born to vaccinated mothers, for different mean durations of infant immunity.

**Duration) (days)**	**Median IRR**	**IQR**
90	1.26	1.23–1.30
182	1.31	1.27–1.36
230	1.33	1.31–1.35
